# The real-world effectiveness and safety of fingolimod in relapsing-remitting multiple sclerosis patients: An observational study

**DOI:** 10.1371/journal.pone.0176174

**Published:** 2017-04-28

**Authors:** Guillermo Izquierdo, Fátima Damas, Maria Dolores Páramo, Juan Luis Ruiz-Peña, Guillermo Navarro

**Affiliations:** Servicio de Neurología, Hospital Universitario Virgen Macarena, Sevilla, Andalucia, Spain; University of Oxford, UNITED KINGDOM

## Abstract

Fingolimod approval was based mainly on two clinical trials, FREEDOMS and TRANSFORMS, which demonstrated the efficacy and safety of fingolimod in patients with multiple sclerosis (MS). We present an observational study that validates these trials findings in a real-world setting, whereby the effectiveness and safety of fingolimod was assessed in Seville’s’ (Spain) clinical practice.

This retrospective study in MS patients assessed effectiveness (relapses, EDSS, gadolinium-enhancing T1 and new/enlarged T2-weighted lesions): total cohort (n = 249) and stratified according to prior treatment (glatiramer acetate/interferon beta-1 [immunomodulator], natalizumab, naïve), gender, basal EDSS score, basal Gd+ lesions, ARR prior to treatment, age at treatment initiation and number of prior treatments. A multivariante model was used to assess the ARR with baseline characteristics. The safety profile (adverse events [AEs]) was also described.

Fingolimod reduced the annualized relapse rate (ARR) by 75%, 67% and 85% in the total cohort, patients previously treated with immunomodulatory and naïve patients (p<0.0001 all cases). However, patients previously treated with natalizumab kept a constant ARR. The ARR results and the consequent increase in the proportion of relapse-free patients were independent of the age at treatment initiation, number of prior treatments, gender and basal Gd+ lesions. Although fingolimod was effective regardless the basal EDSS score and ARR prior to fingolimod treatment, better outcomes were observed in patients with basal EDSS score <3 (0.2 vs. 0.4; p = 0.0244) and ARR ≥ 2 prior to fingolimod treatment (p = 0.0338). Only the basal EDSS score was association with ARR in the first 24 months of fingolimod treatment in the multivariante model (p = 0.0439). The cumulative probability of disability progression was 20% (month-24) in the total cohort, and was independent from prior treatment, age at treatment initiation, number of prior treatments, gender, basal EDSS score, basal Gd+ lesions and ARR prior to treatment.

The real-world fingolimod benefits observed in this study seem to be similar than those observed in previous clinical trials.

## Introduction

Fingolimod (Gilenya Novartis pharmaceuticals) is a sphingosine 1-phosphate receptor modulator, that inhibits the infiltration of potentially autoreactive lymphocytes from lymph nodes to the central nervous system [1,2]. Fingolimod was approved in 2010 by the Food and Drug Administration (FDA) in U.S. and in early 2011 by the European Medicines Agency (EMA), as the first oral disease modifying therapy (DMT) for reducing the frequency of clinical relapses and delaying disability progression in patients diagnosed with relapsing-remitting multiple sclerosis (RRMS).

Fingolimod efficacy was demonstrated mainly in two clinical trials, the 24-months FREEDOMS and the 12-month TRANSFORMS clinical trials. In these trials fingolimod performed better in the clinical and radiological endpoints compared to placebo (FREEDOM trial) and intramuscular interferon beta-1a (TRANSFORM trial) [3,4]. The benefits of fingolimod in the long-term managements of MS were observed in FREEDOMS trial extensions conducted and other clinical trial extensions [5–7]. However, due to clinical trial limitations, observational studies are important to validate the clinical trial’s findings in a real-world setting.

As fingolimod is a relatively new MS treatment, most of the observational studies that have been published to date, have included only a limited number of patients and/or followed the cohort of patients for a short period of time [8–18]. For example, in Europe only six observational studies have been performed, five studies in Italy and one in Portugal [10,11,15–18], all of which had a limited patient numbers and/or short follow-up periods [10,11,15–18].

Therefore, observational studies that have larger patient enrollment numbers and longer follow-up durations are needed in order to corroborate the fingolimod benefits observed in clinical trials. Thus, the aim of this study was to assess the effectiveness and safety of fingolimod in a clinical practice setting in Seville, south-west Spain.

## Materials and methods

### Patients

This was a retrospective study of adult patients diagnosed with MS and who had been treated with fingolimod 0.5 mg/day up to March 2015, at the Hospital Universitario Virgen de Macarena (Seville, Spain). Data was collected in April 2015. This study was conducted according to the Declaration of Helsinki and met Good Clinical Practice guidelines. All patients included in this study provided informed consent and patient records were de-identified and analyzed anonymously. This study together with the informed consents were approved by the Institutional Review Board of the Hospital Universitario Virgen Macarena.

### Measures

Demographic (sex and birth date) and clinical characteristics (MS type, disease duration, Expanded Disability Status Scale [EDSS] score, prior MS treatments) at fingolimod treatment initiation were collected. Additionally, fingolimod effectiveness (treatment duration, number of relapses, EDSS and new gadolinium-enhancing lesions) and safety (adverse events [AEs] and reasons for discontinuation/withdrawal) were collected during fingolimod treatment. The number of relapses was also collected in the 24 months prior to fingolimod treatment initiation.

Disability progression was defined as an increase of at least 1.5 points from an EDSS score of 0, at least 1 point from an EDSS score of between 1 and 5, or at least 0.5 points from an EDSS score greater or equal to 5.5. Disability progression was confirmed on the patient’s next visit according to routine clinical practice (once every 3 months), in the absence of relapse at the time of assessment. The annualized relapse rate (ARR) was assessed in the 24 months prior to fingolimod treatment and during the first 24 months of fingolimod treatment. The number of gadolinium-enhancing (Gd+) T1 lesions and new/enlarged lesions on T2-weighted Magnetic Resonance Imaging (MRI) scans were collected annually. Regarding the safety assessments, patients were under cardiac monitoring after the first dose administration or re-challenge for at least 6 hours, optical coherence tomography exams after the first 3 months, neurological examination every 3 months with EDSS evaluation, and blood test at 1-month, 3-month, 6-month and every 6 months onwards.

### Statistical analysis

Analysis was performed with all the patients who received at least one dose of fingolimod. Effectiveness analyses were performed for the total cohort and stratified according to the prior treatment (glatiramer acetate or interferon beta-1 [immunomodulators], natalizumab and no treatment [naïve]), sex, age at fingolimod treatment initiation (≤ 30 vs. 30 years old), EDSS basal score (<3 vs. ≥3), basal Gd+ lesions (absence vs. presence), ARR prior fingolimod treatment (<2 vs. ≥ 2) and number of prior treatments (1 vs. ≥ 2). Similarly, this analysis was also performed for according to the washout period duration (WPD) in patients who had received natalizumab.

Description of the qualitative variables were performed using absolute (i.e. counts) and relative (i.e. percentages) frequencies while quantitative variables using mean (standard deviation [SD] or 95% confidence interval [95%CI]) and/or median (interquartile range [IQR]) values. Quantitative variables were compared using the Wilcoxon Signed-Rank Test in the case of paired data and the Kruskal-Wallis Test in the case of independent data. Time to first relapse, confirmed progression of disability, new gadolinium-enhancing lesions, disease activity (relapse or confirmed disability progression), 3-component evident disease activity (disease activity or MRI activity), and discontinuation/ withdrawal were evaluated using Kaplan-Meier analysis, and subgroup comparisons were performed with the Log-rank test. Thus, lost to follow up was censored. Univariate and multivariante models were used to assess the ARR with baseline characteristics. Values of p<0.05 were considered statistically significant. No imputations for missing data were performed. The statistical analysis was performed using SAS (Windows 9.2–SAS Institute, Cary, NC).

## Results

### Study population

A total of 249 patients with MS had received at least one dose of fingolimod of which 211 patients had RRMS and 38 patients had secondary progressive MS (SPMS). Table 1 shows the demographic and clinical characteristics of the RRMS patients treated with fingolimod. Briefly, most of the patients were women (71%), with a mean (SD) age of 37.0 (9.9) years. At the time of data collection the mean (SD) disease duration was 11.5 (6.9) years. The mean (SD) fingolimod treatment duration was 33.1 (27.2) months. There were no sex differences in the demographic and clinical characteristics at fingolimod treatment initiation. By contrast, there were significant differences between naïve patients, patients who had received immunomodulators (IM) and patients who had received natalizumab (NTZ) in the disease duration (mean [SD] in years: IM = 11.8 [6.6], NTZ = 14.8 [6.6], naïve = 7.5 [6.4]), patient age (mean [SD] in years: IM = 36.8 [9.7], NTZ = 41.8 [9.3], naïve = 33.0 [9.6]) and EDSS score (mean [SD]: IM = 3.3 [1.7], NTZ = 3.5 [1.7], naïve = 2.1 [1.5]) (p<0.0001 in all). Naïve patients had therefore significantly shorter disease duration, were younger and had lower EDSS score (Table 1). Demographic and clinical characteristics in the patients with SPMS are shown in S1 Table.

**Table 1:** Demographic and clinical characteristics at fingolimod treatment initiation in the patients with RRMS (n = 211).

	Total cohort (n = 211)	Prior-IM (n = 124)	Prior-NTZ (n = 43)	Naïve (n = 44)
Female patients, n (%)	149 (70.6%)	88 (71.0%)	30 (70.0%)	31 (70.5%)
Disease duration (y), mean (SD)	11.5 (6.9)	11.8 (6.6)	14.8 (6.6)	7.5 (6.4)*
Patient Age at FTY initiation (y), mean (SD)	37.0 (9.9)	36.8 (9.7)	41.8 (9.3)	33.0 (9.6)*
EDSS score at FTY initiation, mean (SD)	3.1 (1.7)	3.3 (1.7)	3.5 (1.7)	2.1 (1.5)*
median (IQR)	2.5 (1.5, 4.5)	3.0 (2.0, 4.5)	3.5 (2.0, 4.5)	1.5 (1.5, 2.5)
Duration of FTY (m), mean (SD)	33.1 (27.2)	30.6 (23.0)	26.1 (12.3)	47.4 (41.1)
median (IQR)	25.9 (17.1, 34.7)	25.2 (17.5, 34.5)	30.1 (16.7, 34.3)	24.9 (16.0, 92.6)
Prior treatments, median (IQR)	1.0 (1.0, 2.0)	1.0 (1.0, 2.0)	2.0 (2.0, 3.0)	0.0 (0.0, 0.0)

FTY, fingolimod; IM, Immunomodulator; NTZ, Natalizumab; SD, standard deviation; IQR, interquartile range; y, years; m, months; EDSS, Expanded Disability Status Scale;

*p<0.0001among three sub-groups

### Effectiveness

#### Annualized relapse rate

**Fig 1:**
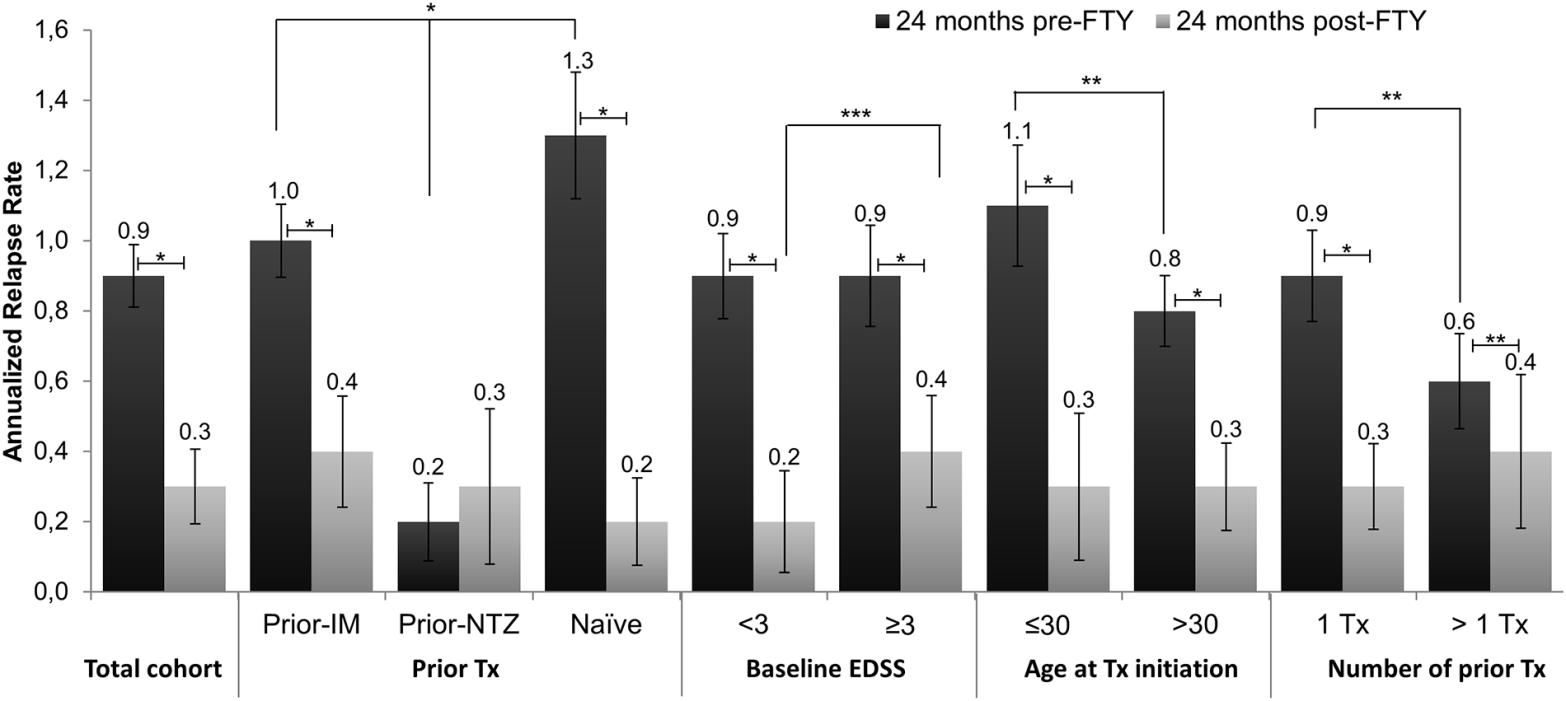
Annualized relapse rate in the 24 months prior to fingolimod treatment and the first 24 months of fingolimod for total cohort and stratified according to prior treatment, sex, age at the fingolimod treatment initiation and number of prior treatments. * p<0.0001; ** p<0.01; *** p<0.05. EDSS, Expanded Disability Status Scale, FTY, fingolimod, IM, immunomodulator; NTZ, natalizumab; Tx, treatment.

For the total cohort, the mean (95% CI) ARR was significant lower during the first 24 months of fingolimod treatment (0.3 [0.2, 0.4]) compared to the 24 months prior to fingolimod treatment (0.9 [0.8, 1.0]) (p<0.0001), with a 67% reduction (Fig 1). Analysis of prior treatment found a significant reduction in the mean (95% CI) ARR of patients who had received immunomodulator (from 1.0 [0.9, 1.1] to 0.4 [0.2, 0.5], 60% reduction) and in naïve patients (from 1.3 [1.1, 1.4] to 0.2 [0.0, 0.3], 85% reduction) (p<0.0001 in both cases), 24 months prior to fingolimod treatment compared 24 months of fingolimod treatment. Conversely, in patients who had received natalizumab, the mean (95% CI) ARR remained similar during fingolimod treatment (0.2 [0.1, 0.3] to 0.3 [0.1, 0.6]; p = 0.1752). These patients treated with natalizumab were treated for an average (SD) time of 34.5 (13.7) months with natalizumab prior to fingolimod and had a mean (SD) washout period duration (WPD) of 4.7 (6.6) months (median = 3.5; IQR = 2.4–4.2). Furthermore, although the mean (95% CI) ARR in the 24 months prior to treatment was significantly different for patients treated with immunomodulator, natalizumab and for naïve patients (p<0.0001), after the first 24 months of treatment the mean ARR were similar among subgroups (p = 0.2400). ARR in patients with SPMS is shown in S2 Table.

A significant reduction of the mean ARR from the 24 months prior fingolimod treatment initiation to the first 24 months of treatment was also observed when patients were stratified according to their age at fingolimod treatment initiation (≤ 30 [n = 56] vs. >30 [n = 155] years), the number of prior treatments (1 [n = 87] vs. >1 [n = 80]), gender (women [n = 149] vs. men [n = 62]), basal EDSS score (<3 [n = 114] vs. ≥3 [n = 81]), basal Gd+ lesions (absence [n = 80] vs. presence [n = 26]) and prior ARR (<2 [n = 188] vs. ≥2 [n = 23]) (p<0.0001 in all the cases except patients with >1 treatment; p = 0.0039). No significant reduction was observed for patients with a WPD of ≤3 months (N = 15) (p = 0.3125) and of >3 months (N = 28) (p = 0.1975). Stratification per age and prior number of treatments showed an ARR reduction of 73% vs. 63%, for patient age ≤ 30 vs. >30 years respectively, and 67% vs. 33% for 1 vs. >1 number of prior treatments respectively. Additionally, an ARR reduction of 67% (from 0.9 [0.8, 1.0] to 0.3 [0.2, 0.4]) in women and 63% (from 0.8 [0.7, 1.0] to 0.3 [0.0, 0.6]) in men was observed. The analysis according to basal Gd+ lesions showed a 67% reduction in ARR in both patients with and without Gd+ lesions at baseline. However no statistically significant differences were observed between these stratified groups (age [p = 0.9693], number of previous treatments [p = 0.5993], gender [p = 0.1292], basal Gd+ lesions [p = 0.9054]). Nonetheless, a statistically significant difference was observed for patients stratified according to basal EDSS score (p = 0.0244) and ARR prior to fingolimod treatment (p = 0.0338). Whereby an ARR reduction of 78% and 56% was observed for patients with basal EDSS score of <3 and ≥3 respectively, and an ARR reduction of 57% and 68% was observed for patients with prior ARR of <2 and ≥2. Therefore, a significantly greater reduction of ARR was seen in patients with an EDSS score of <3 and ARRs prior to fingolimod treatment ≥2. It is worth mentioning that patients with one prior treatment had a higher ARR compared to patients with more than one prior treatment in the 24 months prior to treatment (p = 0.0019). Likewise, patients with ≤ 30 years old had a higher ARR compared to patients with > 30 years old in the 24 months prior to treatment (p = 0.0020). Patients with one prior treatment and with ≤ 30 years old had higher ARR reduction leading to a similar ARR during the first 24 months of treatment (p = 0.5993 and p = 0.9693, respectively). The ARR in the first 24 months of fingolimod treatment was not associated with treatment duration, number of previous treatments, patient age at fingolimod treatment initiation, prior treatments, prior ARR, and basal T1 Gd+ lesions, neither in the univariate nor multivariante models (Table 2). Only basal EDSS score was association with ARR in the first 24 months of fingolimod treatment in both the univariante (p = 0.0138) as well as multivariante (p = 0.0439) models. Thus, patients with EDSS <3 at fingolimod treatment initiation had an ARR reduction of 0.27 points more than patients with EDSS ≥3.

**Table 2:** Multivariant analysis of ARR in the first 24 months of fingolimod treatment with demographic and clinical variants.

	Univariate		Multivariate	
	Estimation	p-value	Estimation	p-value
Gender—Male ^1^	0.0225	0.8510	-0.0357	0.7714
Treatment duration	-0.0069	0.3810	-0.0099	0.2522
Number of previous treatments <2^2^	-0.1964	0.0800	-0.1949	0.1477
Patient age at treatment initiation ≥30 years^3^	0.0418	0.7353	0.0432	0.7610
Basal EDSS <3^4^	-0.2694	0.0138	-0.2399	0.0439
Treatments prior fingolimod treatment			0.16	0.8513*
Immunomodulators ^5^	0.1808	0.1943	0.0897	0.5711
Natalizumab ^5^	0.1753	0.3026	0.0835	0.6992
Basal T1 Gd+ lesions^6^	-0.0770	0.5843	-^‡^	-^‡^
ARR prior fingolimod treatment—<2**^7^	-0.4202	0.0158	-0.2506	0.1715

*Overall p-value of the three variables

** ARR prior fingolimod treatment includes washout period

^‡^ Basal T1 Gd+ lesions was not included in the multivariate model due to the high number of missing.

References used in multivariante analysis:

^1^ Women,

^2^ ≥2 previous treatments,

^3^ Patient age <30,

^4^ Basal EDSS ≥3,

^5^naïve patients,

^6^ No T1 Gd+ lesions and

^7^ ARR prior fingolimod treatment ≥2

**Fig 2:**
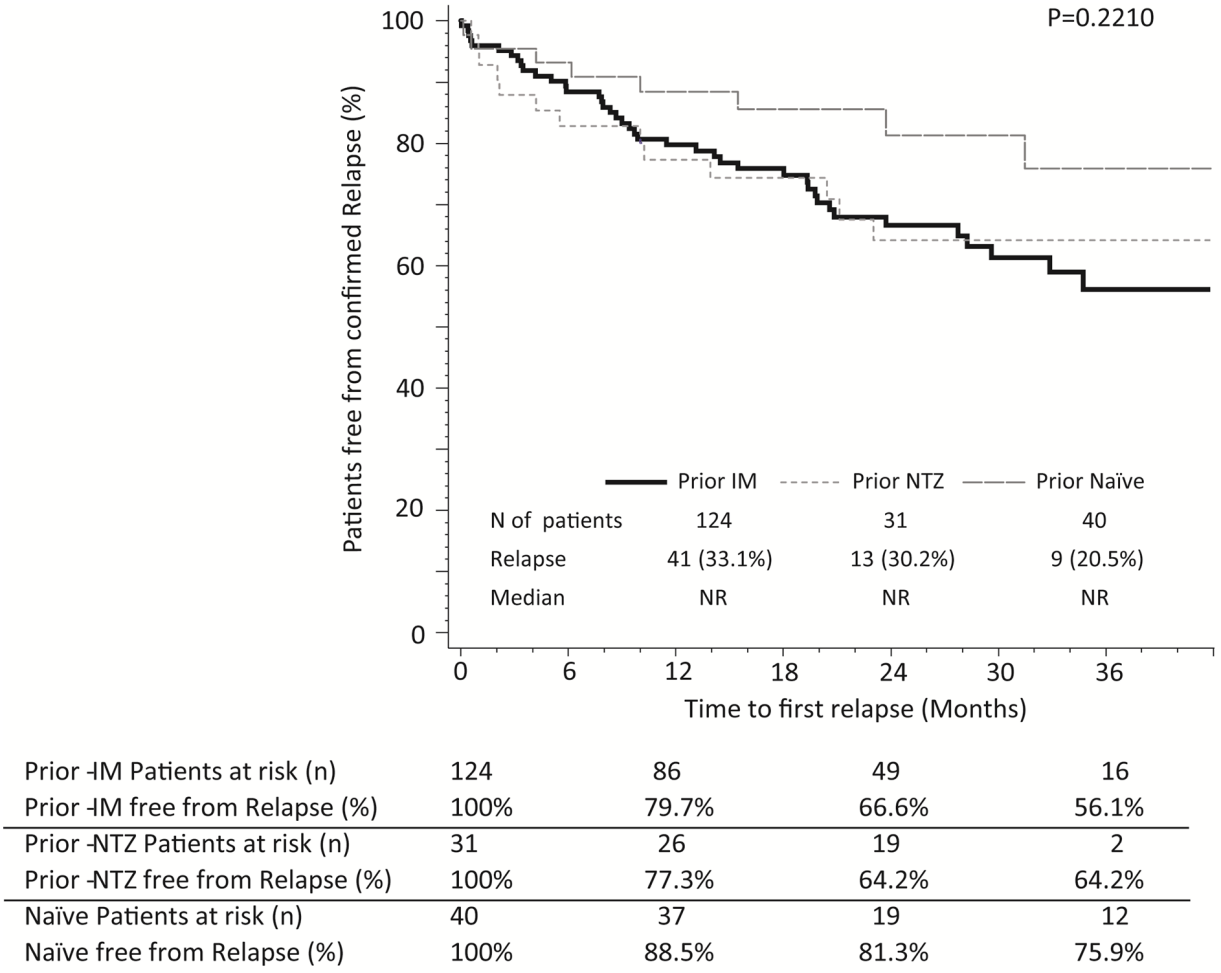
Kaplan–Meier estimates for the time to a first confirmed relapse according to prior treatment. N, number; NR, not reached; IM, immunomodulator; NTZ, Natalizumab.

**Fig 3:**
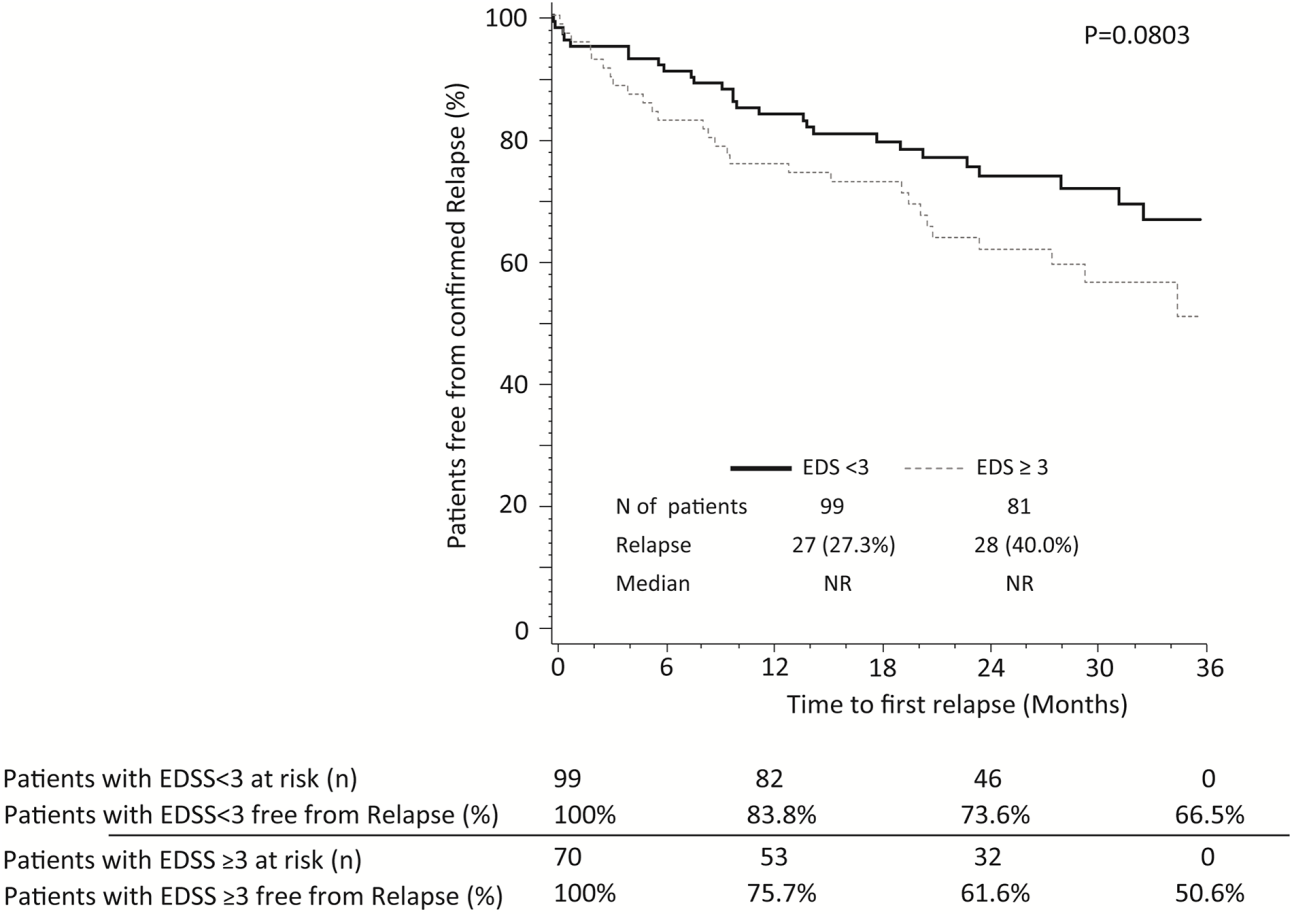
Kaplan–Meier estimates for the time to a first confirmed relapse according to EDSS score. N, number; NR, not reached.

The proportion of relapse-free patients was 81% at month-12, 69% at month-24 and 62% at month-36. No differences were observed in the median time to first relapse according to prior treatment (p = 0.2210) (Fig 2), age at treatment initiation (p = 0.7839), gender (p = 0.1358), number of prior treatments (p = 0.8431), basal Gd+ lesions (p = 0.6869), ARR prior fingolimod treatment (p = 0.1814) or WPD (p = 0.7788) (data not shown). A tendency was observed towards longer time to first relapse in patients with a basal EDSS <3 (p = 0.0803). The number of relapse for patients with basal EDSS of <3 and ≥3 was 27 [27%] and 28 [40%], respectively (Fig 3). Information of relapse in patients with SPMS is shown in S3 Table.

#### Disability

**Fig 4:**
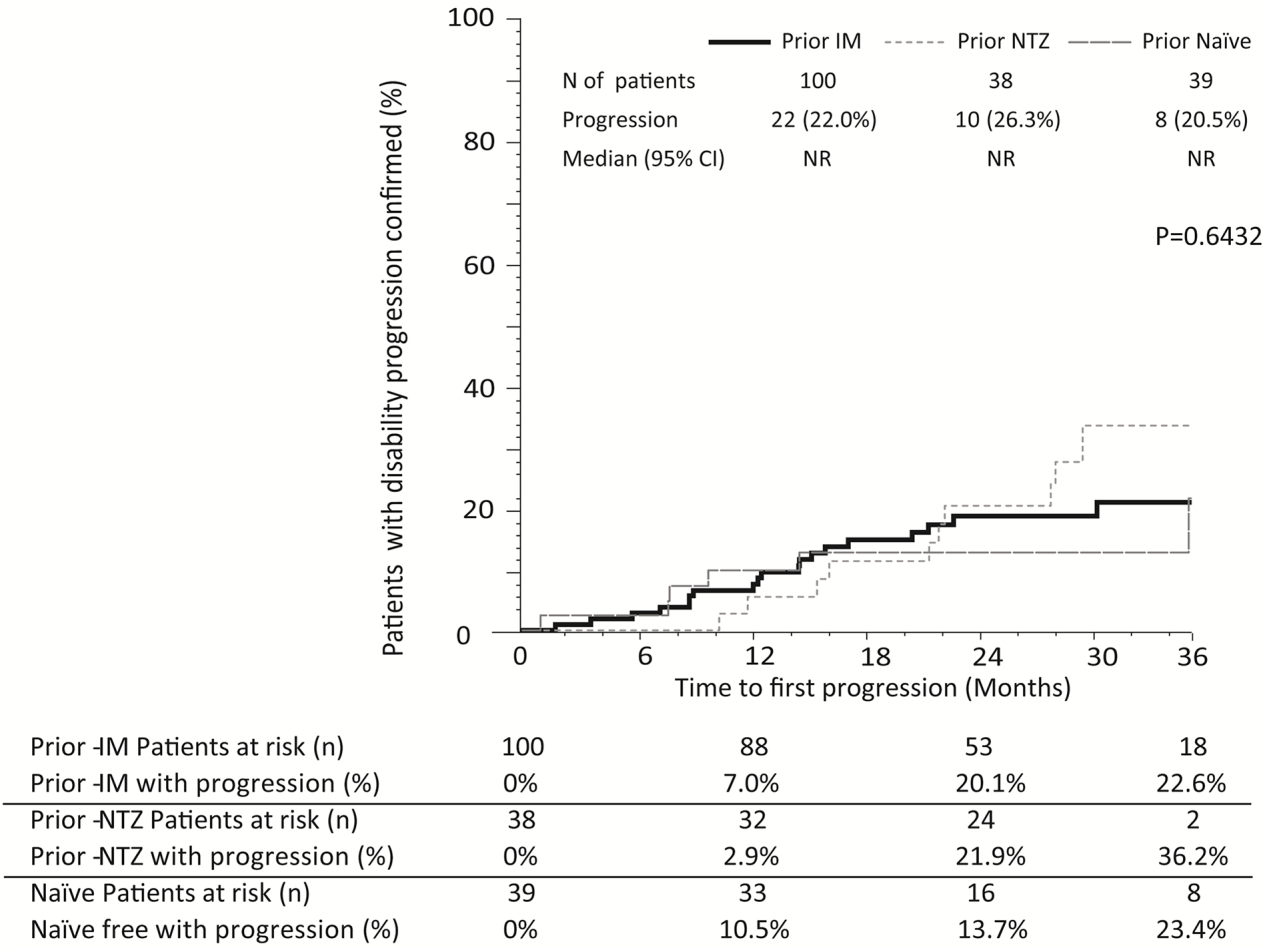
Kaplan–Meier estimates for the time to confirmed disability progression according to prior treatment. N, number; NR, not reached; IM, immunomodulator; NTZ, Natalizumab; 95%CI, 95% confidence interval.

A total of 177 from 211 patients (84%) had an EDSS score during fingolimod treatment that could be confirmed after ≥3 months. A total of 40 (23%) patients suffered at least one confirmed disability progression during fingolimod treatment. The cumulative probability of confirmed disability progression was 6.9% at month-12, 19.5% at month-24 and 27.1% at month-36. The time to confirmed disability progression did not differ according to prior treatment (p = 0.6432) (Fig 4), patient age at treatment initiation (p = 0.3944), number of prior treatments (p = 0.2369), gender (p = 0.2592), EDSS basal score (p = 0.3142), basal Gd+ lesions (p = 0.3784), ARR prior fingolimod treatment (p = 0.9455) or WPD (p = 0.7965) (data not shown). Information of confirmed disability progression in patients with SPMS is shown in S3 Table.

#### MRI-related and other endpoints

Information regarding Gd+ T1 lesions was recorded for 160 from 211 patients (76%) during fingolimod treatment while information of new/enlarged T2-weighted lesions was recorded for 155 from 211 patients (73.5%). A total of 26 (16%) patients reported new Gd+ T1 lesions and 107 patients (69%) reported new/enlarged lesions on T2-weighted images. From the patients with new Gd+ T1 lesions, the proportion of lesions-free patients was 93% at month-12 (mean [SD] number of new lesion = 0.5 [1.5]), 89% at month-24 (mean [SD] number of new lesion = 0.2 [0.9]), and 74% at month-36. While the proportion of patients free of new/enlarged T2-weighted lesions was 69% at month-12, 48% at month-24, and 23% at month-36. MRI-related outcomes in patients with SPMS are shown in S4 Table.

The median (95%CI) time to relapse or confirmed disability progression during fingolimod treatment for the total cohort was not reached. The proportion of patients free for relapse or confirmed disability progression was 74% at month-12, 58% at month-24, and 51% at month-36. The median (95%CI) time to new Gd+ T1 lesions or new/enlarged T2-weighted lesions (MRI activity) during fingolimod treatment for the total cohort was not reached. The proportion of patients free of MRI activity was 87% at month-12, 73% at month-24, and 52% at month-36. The total cohort also reported a median (95%CI) time to relapse, confirmed disability progression or MRI activity of 21.16 (16.07–28.29) months. The proportion of these NEDA-3 patients was 66% at month-12, 46% at month-24, and 36% at month-36.

### Safety

A total of 119 (48%) patients reported at least one AE during treatment. Most of the AEs were considered to be mild in severity. The AEs reported for more than 2% of the patients are displayed in Table 3. The most common AE was lymphopenia; 24% of the patients: 16.1% had levels between 600–400 cells/microliter, 2.8% between 400–200 cells/microliter and 2.8% below 200 cells/microliter. Gamma-Glutamyltransferase (GGT) elevation was reported in 6.8% of the patients: 2.0% less than 3 upper normal limit (UNL), 2.4% between 3–5 UNL and 2.4% more than 5 UNL. Hypertransaminasemia was reported in 3.6% of the patients: 1.6% less than 3 UNL and 2.0% between 3–5 UNL.

**Table 3:** Adverse events (AEs) during fingolimod treatment.

	Total cohort (n = 249)
All AEs, n (%)	
Any AEs	119 (47.8%)
Any AE leading to discontinuation	8 (3.2%)
Most frequently reported AEs (≥2%), n (%)	
Lymphopenia	54 (21.7%)
Gamma-Glutamyltransferase elevation	17 (6.8%)
Hypertransaminasemia	9 (3.6%)
Cardiotoxicity	9 (3.6%)
Infections	9 (3.6%)
Dyslipidemia	7 (2.8%)
Tumor	6 (2.4%)

Forty six (18.5%) patients discontinued/withdrew from fingolimod treatment. The rate of treatment discontinuation/withdrawal at 24- and 36-month was 15.6% and 17.9%, respectively. The main reason for discontinuation was administrative reasons (4.4%), followed by pregnancy or desire for pregnancy (3.6%), AEs (3.2%) and lack of effectiveness (3.2%).

## Discussion

This retrospective study showed that in clinical practice fingolimod reduced the incidence of relapses in patients who switched from immunomodulator and without previous treatment during the first 24 months. Likewise, fingolimod maintained the low incidence of relapses in patients who switched from natalizumab. As a consequence, the ARR was 0.3 and proportion of relapse-free patients after 24 months was 69%, regardless of the prior treatment, age at treatment initiation, number of prior treatments, gender, basal Gd+ lesions or ARR prior to treatment. During the first 24 months, 20% of the patients had disability progression. The time to disability progression was also independent from the prior treatment, age at treatment initiation, number of prior treatments, gender, basal Gd+ lesions and ARR prior to treatment. This study also showed that fingolimod was effective in reducing the ARR regardless the EDSS basal score. However, patients with basal EDSS score of <3 showed better outcomes. The time to disability progression was similar between patients with basal EDSS score of <3 and ≥3.

The effectiveness results obtained in this study were similar to the efficacy results reported in the 0.5 mg/day fingolimod arm in the 12-month TRANSFORM and the 24-months FREEDOMS trials [3,4]: ARR at month-24 (0.18), proportion of relapse-free patients at month-12 (83%) and at month-24 (70%), proportion of patients with confirmed disability progression at month-12 (6%) and at month-24 (13%), proportion of patients with no new/enlarged T2-weighted lesions at month-12 (55%) and at month-24 (51%), proportion of patients new Gd+ lesions-free at month-12 (90%) and at month-24 (90%), and mean number of new Gd+ lesions at month-12 (0.23 [1.0]) and at month-24 (0.2 [0.8]). Therefore, considering the high degree of similarity in the outcomes among the clinical trials and our observational study, along with the pro and cons in the design of both types of studies, the results of this study suggest that the benefits of fingolimod observed in clinical trials are similar to those observed in everyday clinical practice.

Our study found that a basal EDSS score of <3 leads to a higher reduction in ARR. Furthermore, our data suggest that fingolimod has similar benefits in men and in women, as with a similar ARR reduction. However, subgroup analysis performed with the population of FREEDOMS trial observed that men seemed to have a higher relative reduction in ARR than women[19]. More studies are therefore needed to corroborate the conclusions observed in the study hereby presented.

Regarding other clinical practice studies, the results of effectiveness reported here are similar to those obtained with other cohorts of patients from other countries like Germany (PANGAEA study [20]), U.S. (by Hersh et al.[8]) and France (by Nerrant et al. [21]) as well as in other Spanish cities like Madrid (by Galan et al. [22]), validating the results showed here. PANGAEA, a 36-month interim analysis of a prospective study with 3951 patients, at 12 months showed a similar ARR reduction (71%), lower proportion of relapse-free patients (68%) and free of clinical activity (61%). The French cohort of 234 patients with MS (85% RRMS) analyzed by Nerrant et al. showed a 76.0% in ARR reduction. In contrast, the discontinuation rate in this study (25%) was higher than the observed in the present study. Hersh et al. presented a 12-months U.S study, with 306 MS patients, showing similarities regarding the proportion of patients relapse-free (87%) and new Gd+ lesions-free (92%). Unfortunately, Hersh et al. did not report the ARR. Galan et al. studied retrospectively 167 patients and observed an ARR decreased at month-12 of 62%. In this study *naïve* patients and patients with prior immunomodulator use also showed an ARR decreased, but patients with prior natalizumab use kept the ARR after fingolimod treatment initiation. The proportion of relapse-free patients was 81% and of disability progression-free patients was 90%. In light of these data, the results of the current study are in line with the results of other regions like U.S. and Europe.

Regarding the subgroup analysis, our data showed that fingolimod brings a benefit on MS patients regardless of the age at fingolimod treatment initiation, prior treatment, number of prior treatments, gender, basal EDSS score, basal Gd+ lesions, and ARR prior to fingolimod treatment. The few studies available with subgroup analyses usually assessed only the effect of the prior treatment, and our results are in line with the conclusions reached in those studies [10,12,22]. It is worth noting that the reasons for switching to fingolimod or starting fingolimod led to three different patient profiles. Overall, according with the fingolimod Summary of Product Characteristics (SmPC), patients might switch from immunomodulator when they have had a high disease activity despite the immunomodulator treatment, while patients without treatment might initiate fingolimod when they have a rapidly evolving disease in one year [23]. Both are effectiveness reasons for switching treatment. Additionally, patients might switch from natalizumab when they have antibodies against John Cunningham Virus, due to safety concerns. Our data shows that in relapse uncontrolled patients, fingolimod had the capacity to modulate the relapse rate, whereas in previously relapse controlled patients, fingolimod is an option to keep a low relapse rate diminishing the risk of developing progressive multifocal leukoencephalopathy in comparison with natalizumab.

Regarding the safety and tolerability of fingolimod, all the AEs collected were consistent with the safety profiles seen during clinical development and listed in the SmPC [3,4,23]. No tumors diagnosed during treatment seemed to be fingolimod related. A favorable evolution after the specific treatment has been reported for all tumors. Additionally, no birth defects or miscarriage were reported by patients who conceived after fingolimod discontinuation. The proportion of patients who discontinued was similar to both clinical trials (FREEDOM and TRANSFORM) and other clinical studies [3,4,8–10] and only approximately 6% of the patients were observed to lack treatment effectiveness or have poor tolerance.

This study has both strengths and limitations. The clearest limitation is the retrospective nature of the study. One of the biggest strengths of this study is the large sample size and the high number of naïve patients. To our knowledge, up to date, this is the largest observational study carried out in Europe with the highest proportion of naïve patients [8–10,12].

## Conclusion

Fingolimod treatment aims to control the ARR in MS patients. The benefits of fingolimod treatment are independent of the prior treatment, age at fingolimod treatment initiation, number of prior treatments, gender, basal Gd+ lesions and ARR prior to fingolimod treatment. Nevertheless, although fingolimod benefits MS patients regardless the EDSS score at the treatment initiation, our data suggest that patients with basal EDSS score of <3 might have better outcomes. Fingolimod was shown to have a good safety and tolerability profile. Approximately, one in sixteen patients who initiated fingolimod treatment, discontinued for safety or effectiveness reasons. Overall, the real-world benefits observed in this study seem to be similar than those observed in previous clinical trials.

## Supporting information

S1 Table Demographic and clinical characteristics at fingolimod treatment initiation in the patients with SPMS. (DOC)

S2 Table Annualized relapse rate in patients with SPMS. (DOC)

S3 Table Relapses and EDSS scores in patients with SPMS. (DOC)

S4 Table MRI-related outcomes in patients with SPMS. (DOC)
