# Supplementary material for: The real-world effectiveness and safety of fingolimod in relapsing-remitting multiple sclerosis patients: An observational study
Source: PLoS One. 2017 Apr 28;12(4):e0176174. doi: 10.1371/journal.pone.0176174 (PMC5409154; doi:10.1371/journal.pone.0176174)
Supplement: S1 Table — (DOC) [file pone.0176174.s001.doc]

**S1** **Table** Demographic and clinical characteristics at fingolimod treatment initiation in the patients with SPMS

|  | Total cohort  (n=38) | Prior-IM  (n=24) | Prior-NTZ  (n=12) | Naïve  (n=1) | Men  (n=15) | Women  (n=23) |
| --- | --- | --- | --- | --- | --- | --- |
| Female patients, n (%) | 23 (60.5%) | 14 (58.3%) | 7 (58.3%) | 1 (100.00%) |  |  |
| Disease duration (y), mean (SD) | 18.3 (6.4) | 17.5 (6.7) | 19.2 (5.5) | 28.0 () | 18.0 (6.4) | 18.4 (6.5) |
| Patient Age at FTY initiation (y), mean (SD) | 43.3 (8.0) | 44.1 (8.8) | 43.1 (5.9) | 38.0 () | 42.1 (8.1) | 44.0 (8.0) |
| EDSS score at FTY initiation, mean (SD) | 5.9 (0.8) | 5.7 (0.9) | 6.3 (0.7) | - | 5.8 (0.7) | 5.9 (0.9) |
| median (IQR) | 6.0 (5.5, 6.3) | 5.5 (5.5, 6.0) | 6.0 (6.0, 6.5) | - | 5.8 (5.5, 6.0) | 6.0 (5.5, 6.5) |
| Duration of FTY (m), mean (SD) | 18.3 (6.4) | 24.7 (11.8) | 27.3 (8.1) | 65.9 () | 22.5 (14.7) | 29.7 (9.9) |
| median (IQR) | 18.0 (13.0, 22.0) | 27.6 (15.9, 31.8) | 29.8 (21.5, 33.4) | 65.9 (65.9, 65.9) | 16.2 (12.0, 34.7) | 29.7 (26.0, 33.2) |
| Number of Prior treatments, median (IQR) | 2.0 (2.0, 3.0) | 2.0 (1.0, 2.5) | 2.0 (2.0, 3.0) | 0.0 (0.0, 0.0) | 2.0 (2.0, 3.0) | 2.0 (1.0, 3.0) |
| FTY, fingolimod; IM, Immunomodulator; NTZ, Natalizumab; SD, standard deviation; IQR, interquartile range; y, years; m, months; EDSS, Expanded Disability Status Scale | | | | | | |
|  | | | | | | |
