# Supplementary material for: The real-world effectiveness and safety of fingolimod in relapsing-remitting multiple sclerosis patients: An observational study
Source: PLoS One. 2017 Apr 28;12(4):e0176174. doi: 10.1371/journal.pone.0176174 (PMC5409154; doi:10.1371/journal.pone.0176174)
Supplement: S2 Table — (DOC) [file pone.0176174.s002.doc]

**S1 Table** Annualized Relapse rate in patients with SPMS

|  | 24 Months prior to FTY | 24 Months post FTY | p-Value^†^ |
| --- | --- | --- | --- |
| Annualized Relapse Rate, mean (95% CI) |  |  |  |
| Total (n=38) | 0.5 (0.3, 0.8) | 0.1 (0.0, 0.2) | <0.0001 |
| Prior-IM (n=24) | 0.8 (0.5, 1.0) | 0.1 (0.0, 0.3) | <0.0001 |
| Prior-NTZ (n=12) | 0.1 (-0.01, 0.2) | 0.1 (-0.01, 0.3) | >0.999 |
| Naïve (n=1) | 1.5 | 0.0 |  |
| p-Value* | 0.0013 | 0.8637 |  |
| 0-1 prior treatments to FTY (n=4) | 1.1 (0.7, 1.5) | 0.1 (-0.3, 0.5) | 0.1250 |
| >1 prior treatments to FTY (n=33) | 0.4 (0.2, 0.7) | 0.1 (0.0, 0.2) | 0.0008 |
| p-Value* | 0.0088 | 0.8014 |  |
| Women (n=15) | 0.6 (0.3, 1.0) | 0.1 (0.0, 0.3) | 0.0273 |
| Men (n=23) | 0.4 (0.2, 0.6) | 0.1 (0.0, 0.2) | 0.0021 |
| p-Value* | 0.4400 | 0.5059 |  |
| FTY, fingolimod; IM, Immunomodulator; NTZ, Natalizumab  *Between groups in the same point-time  †Between 24 Months prior to FTY and 24 Months post FTY in the same group | | | |
