# Supplementary material for: The real-world effectiveness and safety of fingolimod in relapsing-remitting multiple sclerosis patients: An observational study
Source: PLoS One. 2017 Apr 28;12(4):e0176174. doi: 10.1371/journal.pone.0176174 (PMC5409154; doi:10.1371/journal.pone.0176174)
Supplement: S3 Table — (DOC) [file pone.0176174.s003.doc]

**S1 Table** Relapses and EDSS scores in patients with SPMS

|  | Total cohort  (n=38) | Prior-IM  (n=24) | Prior-NTZ  (n=12) |
| --- | --- | --- | --- |
| Total | 38 | 24 | 12 |
| Relapses, n (%) | 10 (26.3%) | 5 (20.8%) | 4 (33.3%) |
| Time to relapse (m), median | NR | NR | NR |
| Relapse-free survival , % |  |  |  |
| 0 months | 100 (100 - 100) | 100 (100 - 100) | 100 (100 - 100) |
| 12 months | 94.7 (80.6 - 98.7) | 91.67 (70.6 - 97.9) | 100 (100 - 100) |
| 24 months | 76.5 (56.5 - 88.2) | 79.91 (53.9 - 92.2) | 66.7 (28.2 - 87.8) |
| 36 months | 53.4 (25.6 - 74.9) | 53.28 (9.5 - 84.5) |  |
| Total | 30 | 19 | 10 |
| Confirmed Disability progression, n (%) | 13 (43.3%) | 8 (42.1%) | 4 (40.0%) |
| Time to confirmed disability progression (m), median (95% CI) | 23.4 (16.0 - NR) | NR | NR |
| Disability progression-free survival , % |  |  |  |
| 0 months | 100 (100 - 100) | 100 (100 - 100) | 100 (100 - 100) |
| 12 months | 86.7 (68.3 - 94.8) | 89.5 (64.1 - 97.3) | 80.00 (40.9 - 94.6) |
| 24 months | 49.7 (29.0 - 67.4) | 50.3 (24.1 - 71.8) | 54.86 (18.7 - 80.6) |
| 36 months | 49.7 (29.0 - 67.4) | 50.3 (24.1 - 71.8) | ( - ) |
| IM, Immunomodulator; NTZ, Natalizumab; m, months; NR, not reported | | | |
