# Supplementary material for: The real-world effectiveness and safety of fingolimod in relapsing-remitting multiple sclerosis patients: An observational study
Source: PLoS One. 2017 Apr 28;12(4):e0176174. doi: 10.1371/journal.pone.0176174 (PMC5409154; doi:10.1371/journal.pone.0176174)
Supplement: S4 Table — (DOC) [file pone.0176174.s004.doc]

**S1 Table** MRI-related outcomes in patients with SPMS

|  | Gadolinium-enhancing T1lesions | New or enlarged T2 lesions | Gadolinium-enhancing T1lesions or New or enlarged T2 lesions |
| --- | --- | --- | --- |
| Total | 20 | 19 | 20 |
| Relapses, n (%) | 1 (5.0%) | 14 (73.7%) | 14 (70.0%) |
| Time to relapse (m), median | NR | 17.9 (13.4 - 26.3) | 21.0 (13.4 - 26.5) |
| Relapse-free survival , % |  |  |  |
| 0 months | 100 (100 - 100) | 100 (100 - 100) | 100 (100 - 100) |
| 12 months | 100 (100 - 100) | 84.2 (58.7 - 94.6) | 85.0 (60.4 - 94.9) |
| 24 months | 100 (100 - 100) | 34.0 (13.0 - 56.6) | 38.0 (16.2 - 59.7) |
| 36 months | 91.7 (53.9 - 98.8) | 0.00 ( - ) | 11.4 (0.9 - 36.6) |
| IM, Immunomodulator; NTZ, Natalizumab; m, months; NR, not reported | | | |
